# Morning-evening type and burnout level as factors influencing sleep quality of shift nurses: a questionnaire study

**DOI:** 10.3325/cmj.2011.52.527

**Published:** 2011-08

**Authors:** Ayten Demir Zencirci, Sümeyye Arslan

**Affiliations:** 1 Ankara University Health Sciences Faculty, Nursing Department, Ankara, Turkey; 2 Pamukkale University, Denizli School of Health, Denizli, Turkey; Demir Zencirci et al: Morning-evening type and burnout level as factors influencing sleep quality of shift nurses

## Abstract

**Aim:**

To assess the relationship between sleep quality and demographic variables, morning-evening type, and burnout in nurses who work shifts.

**Methods:**

We carried out a cross-sectional self-administered study with forced choice and open-ended structured questionnaires – Pittsburg Sleep Quality Index, Morningness-eveningness Questionnaire, and Maslach Burnout Inventory. The study was carried out at Gazi University Medicine Faculty Hospital of Ankara on 524 invited nurses from July to September 2008, with a response rate of 89.94% (n = 483). Descriptive and inferential statistics were applied to determine the risk factors of poor sleep quality.

**Results:**

Most socio-demographic variables did not affect sleep quality. Participants with poor sleep quality had quite high burnout levels. Most nurses who belonged to a type that is neither morning nor evening had poor sleep quality. Nurses who experienced an incident worsening their sleep patterns (*P* < 0.001) and needlestick or sharp object injuries (*P* = 0.010) in the last month had poor sleep quality. The subjective sleep quality and sleep latency points of evening types within created models for the effect of burnout dimensions were high.

**Conclusions:**

Nurses working consistently either in the morning or at night had better sleep quality than those working rotating shifts. Further studies are still needed to develop interventions that improve sleep quality and decrease burnout in nurses working shifts.

Nurses have to be accessible to patients on a twenty-four hour basis, which is only possible by shift work (1). Shift work puts nurses under stress and, thereby, aggravates their health, well-being, and lifestyle. Furthermore, it worsens natural human circadian rhythm and sleep quality (2) and causes more sleep problems among nurses working rotating shifts than those working non-rotating shifts (3-5).

Also, people working rotating shifts had a variety of other health problems – gastrointestinal problems such as digestive disorders and ulcer, neck and back pain, fatigue, depression, tiredness, job stress, emotional disorders, and an increased risk of cardiovascular diseases (6-8). Sleeping at day-time because of shift system decreased sleep duration and rapid eye movement period (9). Strained nurses more likely experienced depression, somatic disturbances, sleep disorders, and burnout, all of which threaten the quality of health care they provide (1,7,10). Shift workers are often tired, irritated, nervous, and sleepy because of irregular work schedules (8). Over-tiredness also causes concentration loss, which increases errors and accidents (5,9). Furthermore, scarce sleep shortens situational awareness, problem-solving capacity, and vigilance, even further jeopardizing patient safety (1,5).

Nocturnal shift work alters the timing of light exposure and induces circadian rhythm disruption (2,6). Sleep is also affected by body temperature, blood pressure, heart beat rate, light, age, sex, eating patterns, tea or coffee intake, social patterns, circulatory/cardiovascular problems, mental health problems, tobacco, alcohol, having children, being single parent, having a family, and many other factors (10,11). Also, when nurses in hospitals work 12 or more hours for a longer period of time, errors and near errors at work are more likely to occur (12). Mistakes as a result of sleeping problems are most common early in the morning (13).

Some studies investigated how circadian type influenced the performance of individuals (14). The circadian types are morning-type, evening-type, and intermediate type. Morning-types go to sleep early and wake up early, while evening-types are active during the night and cannot get up early (15,16). The shift of the nurses should be adjusted to their living and especially sleeping habits, which is unfortunately currently not the case.

Burnout is another important problem for nurses working shifts (17). It is a psychological response to chronic emotional and interpersonal job-related stressors, and is defined by the three dimensions – emotional exhaustion, depersonalization, and reduced personal accomplishment (18,19). It seems that burnout and sleep quality may affect each other. Although there is no sleep study on nurses, Grossi et al have recently demonstrated the relationship between burnout scores and sleeplessness in women (20).

The aim of this study was to assess the relationship between sleep quality and demographic variables such as arterial blood pressure, body mass index, heart beat rate, tea and coffee consumption, the relationship between sleep quality and morning-evening type, and the relationship between sleep quality and burnout in shift-worker nurses.

## Methods

### Design and setting

A cross-sectional study was carried out between July and September 2008 by a self-administered, forced choice, and open-ended structured questionnaire at Gazi University Medicine Faculty Hospital, Ankara, Turkey. The research population comprised all 637 nurses working in the hospital with a bed capacity of 1085. We excluded the nurses diagnosed with psychotic, neurological, metabolic, and sleep disorders, as well as nurses who either worked less than one year or who were on vacation or on medical leave during July and September 2008. Therefore, 524 nurses were invited to participate, but 26 refused and 15 did not complete the questionnaires, which left 483 nurses who were interviewed, with an overall 89.94% (483/524) response rate.

Nurses in the hospital worked 8-hour shifts from 08.00-16.00, 16.00-24.00, and 24.00-08.00; or 16-hour shifts from 16.00-08.00 and 08.00-24.00, working on average 40 hours or 48 hours per week.

### Participants

The study included 483 female nurses (mean age = 30.41 ± 5.7) with the following characteristics (mean ± standard deviation or median [range]): work experience – 8 (1 to 30) years; systolic blood pressure – 100 ± 12.33 mm Hg; diastolic blood pressure – 65.07 ± 9.21 mm Hg; heart beat rate – 82.23 ± 10.33; body mass index (BMI) – 22.67 ± 3.50; daily teacup – 1.00 (0 to 12); daily coffee cup – 3.00 (0 to 10). Most respondents were married (52.2%) and worked as an in-patient nurse on wards (48.4%). Of those nurses, 65.2% (n = 315) worked rotating shifts (morning-evening-night shift), 5% (n = 24) continuously worked night shifts (evening or night shift), and 29.8% (n = 144) continuously worked day shifts.

Of the nurses, 65% (n = 314) were graduates from Nursing School with baccalaureate degree, 21.9% (n = 106) from Health Services Vocational School Nursing Program with associate degree, and 13% (n = 63) from Vocational Health High School Nursing Program. Health Services Vocational School Nursing Program provides 2 years of nursing education to students who had finished 11 years of basic education. Vocational Health High School Nursing Departments provide 4 years of professional training to students who had finished 8 years of basic education. With 2007 changes in nursing institutions, nursing education in Turkey is now provided only by the universities with baccalaureate degrees. Other nursing schools were closed down.

### Pilot trial

For pilot trial, the first part of the question-form was given to 20 nurses who worked in another hospital and were not a part of the main research population. The pilot trial provided a test of comprehensibility and clarity of the questions, and based on it, self-administered, closed-ended, structured questionnaire interview was revised.

### Ethical considerations

Hospital ethics committee did not require an ethical approval for the study since it included no invasive practices for humans or animals. Instead, hospital management provided written approvals. The author contacted the chief nurse in each ward, explained the purpose of the study, and obtained a verbal permission. The participants signed the informed consent, after they had been informed in detail on characteristics and aim of this study.

### Data collection

We collected data from nurses by using 4 anonymous self-administered questionnaires. Personal Information Form with forced choice and open-ended questions was designed by the authors to obtain information about demographic, socio-economic, individual, and work-related issues that were supposed to have effects on the scores of Pittsburg Sleep Quality Index (PSQI), Morningness-eveningness Questionnaire (MEQ), and Maslach Burnout Inventory (MBI) (Table 1). Height, weight, arterial blood pressure and heart beat rate measurements of participant nurses were recorded in the first visit. Then, nurses were asked to fill in the Personal Information Form, PSQI, MEQ and MBI questionnaires, which were collected at the end of the shift. However, the questionnaires from the nurses who were not able to turn them in because of workload were collected as soon as completed, some even in the same shift.

**Table 1:** Comparison of nurses with good (n = 101) and poor (n = 382) sleep quality according to the Pittsburg Sleep Quality Index (PSQI)

	Sleep quality(mean ± standard deviation or count and percentage)						
Variables	good (PSQI<5)		poor (PSQI≥5)			Statistics value	*P*
Age	30.97 ± 6.18		30.26 ± 5.65			1.041*	0.299
Years employed in nursing	9.33 ± 6.60		8.55 ± 6.38			1.019*	0.308
Body mass index	22.46 ± 2.99		22.73 ± 3.63			0.767*	0.444
Systolic blood pressure	99.85 ± 13.14		100.49 ± 12.12			0.441*	0.660
Diastolic blood pressure	64.25 ± 7.66		65.28 ± 9.58			1.137*	0.257
Heart beat rate	82.13 ± 9.52		82.25 ± 10.55			0.111*	0.912
Daily cup of tea (number)	3.39 ± 2.02		3.76 ± 2.52			0.857^†^	0.392
Daily cup of coffee (number)	0.90 ± 1.54		0.82 ± 1.45			0.314^†^	0.754
Emotional exhaustion	14.58 ± 6.34		19.17 ± 6.64			6.394*	<0.001
Depersonalization	3.41 ± 2.61		5.10 ± 3.37			4.583^†^	<0.001
Personal accomplishment	22.03 ± 3.85		20.50 ± 3.86			3.53*	0.001
Marital status:							
married	56 (55.4)		196 (51.3)			0.548^‡^	0.459
single	45 (44.6)		186 (48.7)				
Children:							
yes	40 (39.6)		150 (39.3)			0.004^‡^	0.951
no	61 (60.4)		232 (60.7)				
Work pattern of nurses:							
continually day shift	37 (36.6)		107 (28.0)			2.898^‡^	0.235
continually night shift	5 (5.0)		19 (5.0)				
rotating shift	59 (58.4)		256 (67.0)				
Resting during the night shift (n = 339):							
yes	32 (50.0)		95 (34.5)			5.293^‡^	0.021
no	32 (50.0)		180 (65.5)				
Status of nurses:							
manager nurse	14 (13.9)		36 (9.4)			4.242^‡^	0.236
in-patient nurse	43 (42.6)		191 (50.0)				
intensive care nurse	30 (29.7)		120 (31.4)				
out-patient nurse	14 (13.9)		35 (9.2)				
Events affecting the sleep pattern during the last month:							
yes	10 (9.9)		119 (31.2)			18.429^‡^	<0.001
no	91 (90.1)		263 (68.8)				
Sleepy in shift:							
yes	63 (62.4)		292 (76.4)			8.111^‡^	0.004
no	38 (37.6)		90 (23.6)				
Sharp object or needle injuries during the last month:							
yes	3 (3.0)		44 (11.5)			5.707^§^	0.017
no	98 (97.0)		338 (88.5)				
Morningness-eveningness type:							
morning type	19 (18.8)		51 (13.4)			8.252^‡^	0.016
neither type	78 (77.2)		279 (73.0)				
evening type	4 (4.0)		52 (13.6)				
Late arrival to work due to waking up late during the last month:							
yes	5 (5.0)			77 (20.2)	12.048^§^		0.001
no	96 (95.0)			305 (79.8)			
Types of home:							
house/apartments	93 (92.1)			334 (87.4)	1.259^§^		0.262
dormitory		8 (7.9)		48 (12.6)			
Having a private room:							
yes		95 (94.1)		336 (88.0)	2.493^§^		0.114
no		6 (5.9)		46 (12.0)			
Appropriateness of room for sleeping:							
yes		98 (97.0)		331 (86.6)	7.654^§^		0.006
no		3 (3.0)		51 (13.4)			
Able to sleep after night shift:							
yes		87 (86.1)		277 (72.5)	7.270^§^		0.007
no		14 (13.9)		105 (27.5)			
A roommate or bed partner:							
no roommate or bed partner		28 (27.7)		118 (30.9)	3.135^‡^		0.371
having a bed partner or roommate in the other room		2 (2.0)		20 (5.2)			
a partner but beds are separate		13 (12.9)		54 (14.1)			
a bed partner		58 (57.4)		190 (49.7)			
Snoring loudly (n = 343):							
never		54 (74.0)		174 (64.4)	6.669^‡^		0.083
fewer than once a week		14 (19.2)		47 (17.4)			
once or twice a week		4 (5.5)		22 (8.1)			
three times or more a week		1 (1.4)		27 (10.0)			
Long pauses between breaths during sleeping (n = 341):							
never		61 (85.9)		201 (74.4)	ELR^║^		0.069
fewer than once a week		7 (9.9)		39 (14.4)			
once or twice a week		3 (4.2)		20 (7.4)			
three times or more a week		0 (0.0)		10 (3.7)			
Twitching in legs during sleep (n = 341):							
never		45 (63.4)		128 (47.4)	10.907^‡^		0.012
less than once a week		15 (21.1)		54 (20.0)			
once or twice a week		10 (14.1)		53 (19.6)			
three times or more a week		1 (1.4)		35 (13.0)			
Incompatibility with roommates or partners and confusion during sleeping (n = 337):							
never		59 (86.8)		174 (64.7)	14.99^‡^		0.002
less than once a week		8 (11.8)		48 (17.8)			
once or twice a week		0 (0.0)		29 (10.8)			
three times or more a week		1 (1.5)		18 (6.7)			

*t test.

†Mann-Whitney U test.

‡Pearson χ^2^ test.

§Continuity correction test.

║Exact likelihood ratio value.

### Instruments

*Pittsburg Sleep Quality Index.* PSQI assesses quality and patterns of sleep through self-reported sleep habits over the last month. It is a global measure with seven subscales; subjective sleep quality, sleep latency, sleep duration, habitual sleep efficiency, sleep disturbance, use of sleeping pills, and daytime dysfunction. A global sleep quality score is then obtained by summing the 7 components. Each component scores from 0 (not in the past month) to 3 points (3 or more times per week) and the global score ranges from 0 to 21. A PSQI total score ≥5 indicates poor sleep quality. A score of 5 yielded a diagnostic sensitivity of 89.6% and a specificity of 86.5%, with an internal consistency (α) of 0.83 and test-retest reliability (r) of 0.85 (21).

Ağargün et al (22) carried out its validity and reliability work in Turkey. The Turkish language PSQI had α = 0.804 and test-retest reliability of 0.98. The PSQI contains 24 questions. Of these, 19 were self-rating questions and 18, included in 7 subscales, were taken into consideration at scoring.

### Morningness-eveningness Questionnaire

Circadian types of nurses were assessed with MEQ, developed originally by Horne and Ostberg (15). MEQ establishes five behavioral categories: definitively morning types (score = 70-86), moderately morning types (score = 59-69), neither types (score = 42-58), moderately evening types (score = 31-41), and definitively evening types (score = 16-30). The morning types get up early and go to bed early, while the evening types are active during the night and cannot get up early. Evening types adjust to night shifts easier (16).

Pündük et al (23) carried out MEQ validity and reliability testing in Turkey. The Turkish language MEQ had α = 0.785 and 0.812 for the first and second applications, respectively, and the test-retest reliability coefficient of 0.84. While analyzing the data, we reduced the categories from 5 to 3: morning type (score = 59-86), neither type (score = 42-58), and evening type (score = 16-41) (11,15,23).

*Maslach Burnout Inventory.* Burnout level of nurses was assessed with the MBI, developed originally by Maslach (24). MBI is a tool that evaluates experienced burnout with three components. Emotional exhaustion (EE) component includes 9 items and refers to feelings of being overextended and depleted of one’s emotional and physical resources. Depersonalization (DP) component includes 5 items and refers to a negative, callous, or excessively detached response to various aspects of the job. Personal accomplishment (PA) component has 8 items and refers to feelings of incompetence and a lack of achievement and productivity at work. Each item of MBI has 5 choices ranging from 0 (never) to 4 (always) (24,25).

High scores on EE and DP components and low scores on PA subscale indicate high levels of burnout. Moderate burnout corresponds to moderate scores on each component. Low scores on EE and DP components and high scores on PA component indicate a low burnout (24). In MBI, the score of each component is evaluated separately. The relation between the three dimensions of burnout is not shown with an overall score. Three different scores are calculated for each individual (24,26).

In Turkey, validity and reliability testing was carried out by Ergin (26). The Turkish language MBI had α of 0.83, 0.65, and 0.72 for EE, DP, and PA, respectively. Test-retest reliability values of EE, DP, and PA were 0.83, 0.72, and 0.67, respectively.

### Data analysis

BMI, PSQI, MEQ, and MBI data were analyzed using the SPSS (SPSS Inc., Chicago, IL, USA), version 16, for descriptive and inferential statistics. Statistical power was strengthened by defining poor sleep quality as a score of ≥5 on PSQI (21,22). Comparisons between socio-demographic variables, nursing work characteristics (ie, years employed in nursing, status of nurses, and work pattern), age, BMI, arterial blood pressure, tea/coffee drinking habit, needlestick injures in the last month, MBI, and morning-evening types were carried out by independent sample *t*-tests for continuous variables and Pearson χ^2^ tests for categorical variables. Mann-Whitney U statistic was used as nonparametric test (Table 1).

We tested contributions of demographic characteristics, work related issues, burnout level, and morning-evening type variables to PSQI by binary logistic regression analysis (with enter method, entry criteria *P* ≤ 0.05). The dependent variable was sleep quality (Table 2). Linear regression analysis (adjusted for EE, DP, and PA) was conducted in order to investigate responsive components to individual morning-evening types of the nurses. Before that, PSQI components were separated to ascertain the effect of morning-evening type for each component after dummy variables had been created for MEQ (Table 3). Pearson r bivariate correlations (two tailed) among PSQI and MBI subscale scores were calculated after the data had been split among continual day shift, night shift, and rotating shift (Table 4).

**Table 2:** Significant/nonsignificant risk factors of poor sleep quality (Pittsburg Sleep Quality Index ≥5) determined by multiple logistic regressions (n = 483)

Variables	Odds ratio (95% confidence interval)	*P*
Age	1.03 (0.92-1.15)	0.560
Years employed in nursing	0.96 (0.86-1.07)	0.499
Body mass index	1.04 (0.95-1.14)	0.315
Systolic blood pressure	0.99 (0.96-1.02)	0.726
Diastolic blood pressure	1.01 (0.96-1.05)	0.634
Heart beat rate	0.99 (0.96-1.01)	0.549
Tea	0.99 (0.88-1.12)	0.921
Coffee	0.96 (0.78-0.18)	0.728
Emotional exhaustion	1.06 (1.01-1.11)	0.016
Depersonalization	1.10 (0.99-1.23)	0.068
Personal accomplishment	0.96 (0.89-1.03)	0.335
Morning-evening type:		
morning	Ref	
neither	1.08 (0.53-2.20)	0.821
evening	2.61 (0.69-9.83)	0.154
Alcohol:		
no	Ref	
yes	0.67 (0.30-1.51)	0.343
Arriving late to work:		
no	Ref	
yes	3.40 (1.23-9.38)	0.018
Marital status:		
married	Ref	
single	0.26 (0.05-1.24)	0.093
Work/shift pattern:		
always day shift	Ref	
always night shift	0.47 (0.11-1.90)	0.291
mix shift/rotating shift	0.76 (0.31-1.82)	0.544
Status of nurses:		
out-patient nurse	Ref	
manager nurse	0.89 (0.31-2.5)	0.841
in-patient nurse	1.26 (0.42-3.78)	0.676
intensive care nurses	1.24 (0.39-3.93)	0.704
Pregnancy:		
no	Ref	
yes	1.94 (0.48-7.79)	0.350
Routine exercise:		
yes	Ref	
no	1.25 (0.71-2.20)	0.431
Menopause:		
no	Ref	
yes	1.61 (0.26-9.94)	0.603
Menstrual cycle:		
regular	Ref	
irregular	1.21 (0.48-3.02)	0.677
Events affecting the sleep pattern:		
no	Ref	
yes	3.70 (1.69-8.09)	0.001
Sleepy during the shift:		
no	Ref	
yes	0.26 (0.70-2.28)	0.426
Medication errors:		
no	Ref	
yes	0.93 (0.40-2.18)	0.884
Sharp object or needle injuries:		
no	Ref	
yes	2.64 (0.71-9.75)	0.144
Sleeping after the night shift:		
yes	Ref	
no	1.77 (0.86-3.62)	0.118
Do you have a roommate or bed partner?		
having a bed partner	Ref	
not having any roomate or bed partner	6.24 (1.29-30.08)	0.022
having roommate or bed partner in a separate room	7.26 (0.91-57.42)	0.060
having a partner but in a separate bed	5.31 (0.99-28.26)	0.050

**Table 3:** The effects of morning-evening type on each component of Pittsburgh Sleep Quality Index (PSQI) determined by linear regressions, adjusted by emotional exhaustion (n = 483)*

Components of PSQI	Neither type (95% confidence interval)				Evening type (95% confidence interval)			
	β	lower	upper	*P*	β	lower	upper	*P*
Subjective sleep quality	0.15	-0.01	0.33	0.074	0.40	0.16	0.64	0.001
Sleep latency	0.22	-0.00	0.45	0.059	0.54	0.22	0.87	0.001
Sleep duration	0.03	-0.21	0.28	0.792	-0.15	-0.50	0.19	0.393
Habitual sleep efficiency	0.006	-0.24	0.25	0.964	0.10	-0.24	0.44	0.563
Sleep disturbances	0.04	-0.09	0.19	0.517	-0.05	-0.25	0.15	0.614
Use of sleep medication	0.05	-0.06	0.17	0.390	0.12	-0.03	0.29	0.120
Daytime dysfunction	0.27	-0.15	0.70	0.213	0.08	-0.50	0.67	0.782

*While constructing the dummy variable, morning type is taken as a reference category.

**Table 4:** Correlations between Pittsburgh Sleep Quality Index (PSQI) and Maslach Burnout Inventory components according to work pattern of nurses

Components		Always day-shift nurses (n = 144)			Always night-shift nurses (n = 24)			Rotating shifts nurses (n = 315)		
		EE	DP	PA	EE	DP	PA	EE	DP	PA
Subjective sleep quality	r	0.275	0.222	0.016	0.351	0.467	-0.111	0.306	0.160	-0.105
	*P*	0.001	0.007	0.850	0.093	0.022	0.607	*P* < 0.001	0.005	0.063
Sleep latency	r	0.101	-0.028	-0.022	0.057	0.162	0.071	0.178	0.075	0.001
	*P*	0.226	0.738	0.795	0.791	0.449	0.743	0.002	0.183	0.987
Sleep duration	r	0.211	0.212	0.072	0.120	0.190	-0.025	0.182	0.124	<0.001
	*P*	0.011	0.011	0.394	0.576	0.373	0.909	0.001	0.028	0.989
Sleep efficiency	r	0.237	0.120	-0.084	0.105	-0.103	0.243	0.091	0.099	-0.002
	*P*	0.004	0.154	0.316	0.627	0.631	0.253	0.107	0.079	0.971
Sleep disturbance	r	0.288	0.224	-0.071	0.139	-0.059	-0.125	0.246	0.193	-0.027
	*P*	*P* < 0.001	0.007	0.399	0.518	0.784	0.561	*P* < 0.001	0.001	0.632
Sleep medication	r	0.111	0.023	0.068	0.384	0.583	-0.052	0.067	0.027	0.032
	*P*	0.186	0.784	0.420	0.064	0.003	0.811	0.235	0.634	0.569
Daytime dysfunction	r	0.295	0.308	-0.099	0.421	0.234	0.051	0.224	0.104	-0.066
	*P*	*P* < 0.001	*P* < 0.001	0.235	0.040	0.270	0.815	*P* < 0.001	0.065	0.241
PSQI global score	r	0.185	0.224	-0.032	0.273	0.225	-0.076	0.308	0.196	-0.218
	*P*	0.026	0.007	0.699	0.197	0.291	0.725	*P* < 0.001	*P* < 0.001	*P* < 0.001

*Abbreviations: EE – emotional exhaustion; DP – depersonalization; PA – personal accomplishment.

## Results

The mean ± standard deviation global PSQI value of nurses was 7.32 ± 3.42 and the global PSQI of 79.1% (n = 382) of nurses was ≥5. The mean ± standard deviation total sleep time was 6.95 ± 0.99 hours. Most of the basic socio-demographic variables did not affect sleep quality (Table 1). EE (*P* < 0.001) and DP (*P* < 0.001), as well as PA decreased sleep quality (*P* = 0.001). Neither MEQ type also decreased sleep quality (*P* = 0.016). The events experienced in the last month affecting the sleep pattern (*P* < 0.001), sleepiness during the shift (*P* = 0.004), sharp object and needlestick injuries (*P* = 0.010), and lateness or failure to wake up in time were associated with sleep quality (*P* < 0.001) (Table 1).

Emotional exhaustion, lateness to the work, disturbed sleep pattern in the last month, and having no roommate or no bed-partner were significantly associated with poor sleep quality (Table 2).

Excluding the effect of EE, DP, and PA on sleep quality, we aimed to determine the effect of morning-evening type on PSQI. In order to investigate this, dummy variables were formed according to MEQ and the effect of these variables on PSQI components was identified using linear regression method after having made the necessary corrections according to EE, DP, and PA. In the models created by considering the effect of EE, DP, and PA, subjective sleep quality and sleep latency points of evening types were high (Table 3). In the models created considering the effects of DP and PA, subjective sleep quality (β = 0.45, 95% confidence interval [CI] from -0.22 to 0.69 for DP, and β = 0.49, 95% CI from -0.24 to 0.74 for PA) and sleep latency (β = 0.60, 95% CI from 0.27 to 0.92 for DP, and β = 0.62, 95% CI from 0.29 to 0.94 for PA) points of evening types were high. In the models created by making corrections depending only on PA, sleep latency (β = 0.24, 95% CI from 0.003 to 0.48) increased in neither MEQ type but not in evening type.

Nurses working consistently either in the morning or at night had better sleep quality than those working rotating shifts (Table 4). Nurses working consistently night shifts had better sleep quality than all others.

## Discussion

Most nurses in this study (79.1%) experienced poor sleep quality. This was expected since most of them (65.2%) worked rotating shifts. The finding is in accordance with previous studies (27-30).

Nurses with poor sleep quality had higher burnout levels, experienced something that affected their sleep quality in the last month, were late for work because they could not awake up in the morning, had significantly more sharp object/needlestick injuries, and were sleepy at work. Alimoğlu and Dönmez (31) also found a higher burnout level in nurses with sleep disorders. The strongest predictor of sleep quality in our study was the participant’s natural morningness-eveningness sleep pattern rather than work pattern.

Nurses working rotating shifts experienced more sleeping problems and sleepiness at work than nurses working continuous day/night shifts. Furthermore, nurses in rotating shifts had more accidents or errors. Therefore, rotating shifts should be avoided to assure safety of nurses and patients (32).

Sleep quality and work pattern had a low but significant correlation, but the MEQ type was not clearly associated with poor sleep quality. Admi et al showed that work pattern did not affect lateness, absence, or accidents and errors at work (33). On the other hand, some other studies indicated that sleeplessness or poor sleep quality increased work accidents (34,35). Different results on sleep quality were reported because of factors influencing biopsychosocial integrity of respondents that might affect sleep quality, such as stress and anxiety.

Healthcare workers in the night shifts or those who worked 60 hours a week were found to have higher risk of sharp object or needlestick injuries (36,37), and higher risk errors or near errors (38). Similar results were also found in this study.

Most nurses in the study had difficulty to sleep after the night shift. Bad sleep quality led to twitching in legs, adaptation problems, and confusion during sleep. Other studies also showed that nurses working rotating or night shifts were more tired than others (39) and that sleepiness decreased after they had started working morning shifts only (9).

Our study showed that emotional exhaustion, any incident influencing sleep pattern in the last month, and having no roommate or having no (bed) partner affected sleep quality. Such a finding in individuals with no roommate or bed partner might be explained by their feeling lonely and in need of emotional support. Exhaustion, on the other hand, decreases or diminishes self-confidence and interest in work, as well as causes fatigue and weakness (24). An individual with chronic exhaustion might lose the initiative, grow limited working capacity, and develop a lack of stamina, fortitude, and toughness (25). It has been reported that interns with higher burnout levels experienced chronic sleep deprivation (40).

Morning-evening type and shift pattern of nurses did not affect sleep quality in our study. Chung et al, on the other hand, reported that chronotype affected sleep quality (11). These contradictory results require further studies on the topic. Admi et al found that shift pattern did not affect sleep quality (33). However, some studies showed that rotating shifts worsened circadian rhythm and risked the safety of both health care workers and patients (32,34,35,41,42).

In this study, evening type nurses had poorer subjective sleep quality and sleep latency, which were subscales of PSQI, than nurses of other two types. The administration in the hospital under study did not, however, arrange shifts according to chronotype. Evening type participants were shown to have more negative habits than morning types (14). Morning-types had early sleep schedules and circadian rhythms, and regular waking-, bed-, and sleep-time. Evening types, on the other hand, had late sleep schedules and circadian rhythms, and irregular waking-, bed- and sleep-time. Also, evening-types experienced more common irregular sleep and lifestyle habits, and dissatisfaction with the sleep (14).

Poor sleep quality due to increased burnout level in nurses working rotating shift was an interesting finding, since it might have been expected that nurses working night shifts have lower job stress level than others. Similarly, nurses working fixed day shifts had increased EE and DP scores. Adaptation of biological rhythm would be a lot easier for nurses working fixed shifts than rotating shifts. Although separate studies were available, we did not find any study that investigated MBI, PSQI, and work patterns together. Jamal and Baba (42), for example, found no correlation between work pattern and burnout levels but found higher health problems for nurses working rotating shifts. Newey and Hood studied the relationships of work patterns and sleep/fatigue, and found that night shift was the worst shift followed by day shift and evening shift (41). Gold et al indicated that error rate related to sleepiness of nurses working rotating shift was twice as high as that in other shifts (32).

A limitation of this study is that it was carried out on a given nurse population in Ankara, with a self-reported questionnaire, which might result in a biased reply from each respondent. Therefore, generalizations should be made carefully and be limited only to the investigated population. In conclusion, our findings showed that majority of nurses experienced poor sleep quality and had increased levels of burnout, especially rotating shift nurses. Most of the nurses who did not belong to either morning or evening type had poor sleep quality. Further studies are needed to plan interventions that decrease burnout and improve sleep quality for shift-work nurses. These interventions would likely improve nurses’ overall well-being and working conditions and patients’ safety. The hospital administrations should take workers’ chronotypes into account when forming the shift lists.
